# 

**Published:** 1900-04-21

**Authors:** 


### NOTICE TO CORRESPONDENTS.

All MS., Letters, Books for Review, and other matters intended for the Editor should be addressed The Editor, "The Hospital," The Hospital Building, 28 & 29, Southampton Street, Strand, W.C.
The Editor cannot undertake to return rejected MS., even when accompanied by stamped directed envelope.

**Notice to Advertisers and Subscribers.**

All Advertisements, Orders for Copies of The Hospital, and Business Communications should be addressed to **THE MANAGER (Not to the Editor)**, The Hospital, The Hospital Building, 28 & 29, Southampton Street, Strand, London, W.C.

The Cost of Subscription, post free, is as follows:—Per week, 2½d.; per quarter, 2s. 8d.; per half-year, 5s. 3d.; per annum, 10s. 6d. Foreign:—Per annum, 15s. 2d., payable, in all cases, in advance.

**NOTICE.—Vol. XXVI. of "The Hospital" (April, 1899, to September, 1899), in handsome cloth binding, is now on sale at the Office of this Journal, price 7s. 6d. Cases for binding the Numbers contained in this Volume 1s. 6d. Index to Vol. XXVI., 2½d., post free.**

**The Monthly Part for April is now ready. Price 6d., by post 8d.**

### NOTES ON BOOKS.

We have received another fascicule (the third of the fourth volume) of the splendid Dictionaire de Physiologie by Charles Richet, professor of physiology in the faculty of medicine at the Paris University, now being issued by Baillière et Cie, of Paris. Commencing with the termination of the article on Cyanhydrique (acide), it ends by one on Digestion. The article on Diabetes, by E. Hédon, is especially worthy of notice. That on Defensive Functions, by Charles Richet, is a striking presentment of our present knowledge on this subject.

### THE STUDENT OF MEDICINE.

### APPOINTMENTS.

A. J. Bieneman, M.B.Edin., appointed Medical Officer for the Farnham Royal District of the Eton Union. L. C. Burrell, M.A., M.B., B.C.Camb., appointed Medical Officer for the Kew District of the Richmond Union. C. E. Cooper, M.B., B.C.Camb., appointed Medical Officer for the Second District of the Plympton St. Mary Union. C. E. Cooper, M.B., B C.Camb., appointed Certifying Factory Surgeon for the Urban District of Ivybridge, the civil parish of Ugborough in Totness Rural District, and the civil parishes of Cornwood, Harford, and Ermington in Plympton St. Mary Rural District. J. Jones, L.R.C.P.Edin., L.F.P.S., Glasg., appointed Medical Officer for the Dolgelly District of the Dolgelly Union. Miss B. Knowles, M.B.Lond., appointed Assistant Medical Officer to the Bethnal Green Workhouse. E. T. Martin, M.D., appointed Medical Officer for the Southport District of the Ormskirk Union. G. B. Mason, M.R.C.S., L.R.C.P., appointed Medical Officer for the Sixth District of the Lutterworth Union. J. C. Muir, M.B., B.C.Camb., appointed Senior Assistant Medical Officer at the Crumpsall Workhouse, Manchester. J. Patton, M.B., C.M.Edin., appointed Medical Officer for the Stamford Bridge District of the Pocklington Union. A. Seddon, L.R.C.P., L.R.C.S.Edin., appointed Medical Officer for the Frome District of the Frome Union. A. T. Sissons, M.B., B.Ch.Vict., appointed Second Resident Medical Officer at the Crumpsall Workhouse, Manchester. F. J. Smith, M.D., F.R.C.P Lond., appointed Physician to the National Orthopædic Hospital. J. Wigg, L.R.C.P.Lond., appointed Medical Officer for the Second Ward of the Parish of St. Pancras. M. Young, M.D., Mast. Surg., D.P.H., appointed Medical Officer of Health for the County Borough of Stockport.

### ST. THOMAS'S HOSPITAL MEDICAL SCHOOL.

At the recent examinations, held at the end of the winter session, the following medals and prizes were awarded: The Mead medal to Mr. R. B. Kinloch; the Chiselden medal to Mr. T. H. Edwards; a prize of £10 to each of the following—Mr. F. C. Eve (medicine), Mr. W. H. O. Woods (surgery), Mr. T. S. Taylor (midwifery and diseases of women), Mr. J. E. H. Sawyer (pathology), Mr. J. L. Lock (pharmacology), Mr. R. B. Kinloch (forensic medicine and insanity), Mr. W. B. Fry (public health); the Peacock scholarship (£38 10s.) and a college prize of £20 were divided between Mr. H. S. Bennett and Mr. F. W. W. Smith (second year); the William Tite scholarship (£27 10s.) to Mr. K. Takaki (first year).

Royal 8vo., illustrated by a series of Original Photo-micrographs and many Illustrations in the Text, cloth gilt, 7s. 6d. net,

**THE MENOPAUSE AND ITS DISORDERS.**

With Chapters on Menstruation.

By A. D. LEITH NAPIER, M.D., M.R.C.P.

London: The Scientific Press, Ltd., 28 & 29, Southampton Street, Strand, W.C.

